# Individual Differences in Working Memory Capacity Modulate Electrophysiological Correlates of Semantic Negative Priming From Single Words

**DOI:** 10.3389/fnbeh.2021.765290

**Published:** 2021-11-18

**Authors:** Montserrat Megías, Juan J. Ortells, Isabel Carmona, Carmen Noguera, Markus Kiefer

**Affiliations:** ^1^Department of Psychology, University of Almería, Almería, Spain; ^2^CEINSA, Health Research Center, University of Almería, Almería, Spain; ^3^Department of Psychiatry, Ulm University, Ulm, Germany

**Keywords:** individual differences in working memory capacity, semantic negative priming, event-related potentials, N400 ERP component, late positive complex

## Abstract

In the present study, event-related potentials (ERPs) were registered during a semantic negative priming (NP) task in participants with higher and lower working memory capacity (WMC). On each trial participants had to actively ignore a briefly presented single prime word, which was followed either immediately or after a delay by a mask. Thereafter, either a semantically related or an unrelated target word was presented, to which participants made a semantic categorization judgment. The ignored prime produced a behavioral semantic NP in delayed (but not in immediate) masking trials, and only for participants with a higher-WMC. Both masking type and WMC also modulated ERP priming effects. When the ignored prime was immediately followed by a mask (which impeded its conscious identification) a reliable N400 modulation was found irrespective of participants’ WMC. However, when the mask onset following the prime was delayed (thus allowing its conscious identification), an attenuation of a late positive ERP (LPC) was observed in related compared to unrelated trials, but only in the higher-WMC group showing reliable behavioral NP. The present findings demonstrate for the first time that individual differences in WMC modulate both behavioral measures and electrophysiological correlates of semantic NP.

## Introduction

Working memory (WM) has been defined as the capacity to actively retain and manipulate a limited amount of internal information (e.g., Baddeley, 1986). WM represents a key cognitive resource for many complex cognitive activities in everyday life such as action control or problem solving (Funke, 2010). WM is not only critical for storage and manipulation of information, but also plays a role in maintaining goal-directed behavior in the presence of potential distractors or contextually inadequate alternative responses. To successfully direct our behavior toward task-relevant information, both the target and competing distractors must remain clearly separated in processing. WM has been proposed to be fundamental in this process, and specifically in selective attention, which enhances target processing (e.g., Lavie et al., 2004; Gazzaley and Nobre, 2012).

A variety of studies over the last decades have demonstrated that a reduction in the availability of WM resources is associated with a decreased capacity to inhibit or suppress the processing of irrelevant competing information in selective attention tasks (e.g., Engle and Kane, 2004; Shipstead et al., 2014; Ortells et al., 2016b; Megías et al., 2020).

An experimental paradigm widely used to measure attentional selection, which unlike some other selection tasks (e.g., Flanker, Stroop), allows to investigate the fate of a previously ignored stimulus representation, is that of Negative Priming (NP). The so-called NP effect refers to the demonstration that participants’ reactions to a probe target are slowed down or are more error prone when such stimulus appeared as an ignored distractor on a preceding prime display, compared with a target that did not appear on the prime display (Dalrymple-Alford and Budayr, 1966; Tipper, 1985; see Mayr and Buchner, 2007; Frings et al., 2015, for reviews). NP has typically been observed when the ignored prime is presented along with a simultaneous relevant stimulus to which participants must attend and/or respond. Yet, further work has successfully reported reliable NP even in the absence of distractors on the prime display (i.e., single-NP; e.g., Milliken et al., 1998; Frings and Wentura, 2005; Noguera et al., 2007, 2015; Chao and Yeh, 2008).

An influential theoretical explanation is that NP is a behavioral index of a persisting inhibition mechanism, which would act to suppress (and/or decouple from potential effectors) the activation levels of the ignored (selected-against) prime distractor. The inhibitory action is assumed to *persist* for some time, thus impairing (e.g., delaying) responses to the stimulus when it becomes the relevant to-be-responded target in subsequent probe display (e.g., Tipper, 1985; Tipper and Cranston, 1985; Houghton and Tipper, 1994). Other researchers have explained NP in terms of a backward-acting process of episodic retrieval. In this account, NP would occur when the current probe target triggers retrieval of a previous encounter with the same stimulus, which on that occasion served as an irrelevant prime distractor, causing a delay in the response selection process (e.g., Neill et al., 1992; Rothermund et al., 2005; Mayr and Buchner, 2006; for other alternative accounts see Milliken et al., 1998).

Evidence has accumulated for both accounts, and there are, in fact, some hybrid models conceiving that both forward-acting persisting inhibition and backward-acting retrieval processes would indeed contribute to the NP effect. For example, according to the *distractor inhibition* framework proposed by Tipper (2001; see also Gibbons and Frings, 2010), the main relevant difference between inhibitory and memory retrieval theories relates to the assumption of whether inhibition that is initially allocated to an ignored (selected-against) distractor on the prime display is actively reinstated by a retrieval mechanism during the processing of the probe target, or persists from the prime to the probe display.

In either case, there is converging evidence that the distractor inhibition mechanism supposedly underlying NP depends critically on the availability of cognitive control (WM) resources. A first kind of support comes from research on cognitive aging. Relative to younger adults, older people, who are assumed to have reduced WM capacity (WMC), are disproportionally impaired at attention tasks that require active ignoring of irrelevant information. Thus, they show not only increased interference effect from competing distractors in conflict tasks, but also a reduced (or even absent) NP effect from irrelevant stimuli, indicating that they were unable to efficiently ignore to them (e.g., Mayas et al., 2012; see also Noguera et al., 2019).

Further evidence for a close link between WM and selective attention comes from studies that evaluated the processing of distractors in selective attention tasks (e.g., NP) while varying mental load (e.g., high vs. low load) in a concurrent WM task. The ignored prime distractor usually produces reliable NP only when the concurrent memory task demands are low. In contrast, under a high memory load, the NP effect is often eliminated or even reversed to positive priming (PP), suggesting that the processes that contribute to NP require attentional resources. Similar findings have been reported using a standard NP procedure (i.e., the prime and probe displays contain at least two stimuli; Engle et al., 1995; Conway et al., 1999; De Fockert et al., 2010; Gibbons and Stahl, 2010), as well as single-prime NP paradigms (e.g., Chao and Yeh, 2008; see also Chao, 2011).

Converging support for a dependence of NP on WM control resources comes from studies examining differences in NP between higher-WMC and lower-WMC individuals (i.e., scoring in the upper vs. lower quartiles in several WM and attention control tasks). A consistent result is that only participants having a higher WMC showed reliable NP, whereas lower-WMC participants did not (e.g., Conway et al., 1999; see also Long and Prat, 2002). The differential priming pattern showed by higher vs. lower-WMC individuals has been reported not only with standard identity NP procedures (i.e., the ignored prime is repeated as the probe target), but also using a semantic NP paradigm (i.e., the prime and target stimuli are highly associated members from the same semantic category), which would involve of a more abstract (conceptual) level of representation.

A semantic NP paradigm has been used in a recent study by Ortells et al. (2016b). In this experiment, individuals high and low in WMC (as assessed by their performance in several WM and attention control tasks) were instructed to either attend to or ignore a single prime word that was followed by either a semantically related or unrelated target word, on which participants performed a lexical decision (see also Noguera et al., 2007). Ortells et al. (2016b) found that individual differences in WMC mainly affected the processing of the ignored primes, but not the processing of the attended primes: Whereas a similar positive semantic priming effect (PP) for attended primes was found in both participant groups, reliable semantic NP was shown only by the high WMC group. Yet, the lower-WMC participants showed a PP effect from ignored primes. These findings of reliable NP for high but not for low WMC individuals fit well with the idea that distractor inhibition is resource dependent. Thus, a lower WMC could be associated with a reduced ability of control mechanisms to effectively inhibit the processing of an ignored prime distractor, thus explaining the lack of NP in the low-WMC group.

The modulation of semantic NP by individual differences in WMC has recently been replicated by Megías et al. (2020). In their NP study, participants were instructed to always ignore the single prime word on every priming trial. A second main difference with the Ortells et al.’s (2016a) study is that the ignored prime was followed either after a 314-ms delay or immediately by a pattern mask that remained on the screen until target onset, with both masking delay (delayed vs. immediate) varying randomly from trial to trial.

Several NP studies using similar masking conditions had demonstrated that an ignored single prime lead to reliable semantic NP when the mask onset is delayed (or there is a blank inter-stimulus-interval between the mask offset and the target onset; e.g., Daza et al., 2007; Wang et al., 2014, 2018; see also Noguera et al., 2007). In contrast, the NP effect is completely absent (or even reversed to facilitatory priming) under immediate masking conditions, in which the mask remains on the screen throughout the prime-target interval and thus prevents conscious prime identification (e.g., Daza et al., 2007; Wang et al., 2014, 2018).

Based on a hypothesis originally developed by Houghton et al. (1996) and Wang et al. (2014, 2018) suggest that the presence of a masking pattern that persists at the same spatial location where a target will appear, would generate a continuous perceptual input that could interfere with the implementation of the attentional inhibition resulting from an ignore instruction. By contrast, when the mask onset is delayed, the masking stimulus would not interfere with the buildup of the inhibition, so an ignore instruction could lead to reliable NP.

To the extent that the presentation of a persisting mask indeed interferes with attentional inhibition, Megías et al. (2020) expected to observe a lack of NP in that masking condition for both high-WMC and low-WMC participants. In clear contrast, in the delayed masking condition allowing the implementation of resource-dependent distractor inhibition, the ignored priming pattern should, however, be reliable modulated by WMC. Thus, only participants with a higher-WMC, but not those with a lower-WMC, should show reliable NP in the delayed masking condition. The reliable three-way interaction between Masking Type, Relatedness and WMC, reported by Megías et al. (2020), was clearly consistent with those predictions.

### Current Study

The demonstration that individual differences in WMC can modulate NP at a semantic level of representation, constitutes a relevant finding in the literature.

Most of prior research reporting a dependence of NP on cognitive control (working memory) resources, has used different versions of the identity (or repetition) NP paradigm, in which the same stimulus which appears as a to-be-ignored distractor in a prime display is presented as the to-be-responded target on the subsequent probe display (e.g., Engle et al., 1995; Conway et al., 1999; De Fockert et al., 2010; Gibbons and Stahl, 2010; Mayas et al., 2012).

Identity NP has showed to be a robust behavioral effect, which has been observed for a wide variety of different stimuli, tasks, and populations of participants (see Fox, 1995; Frings et al., 2015, for reviews). Although, it is usually accepted that NP can also rely on the semantic similarity between the ignored prime and the probe target, semantic NP effects have often been weaker and difficult to replicate (especially when words are used as prime stimuli). Semantic NP has proved to be a reliable effect only under several limited conditions, being highly sensitive to several minor procedural differences, such as the type of probe task, the strength with which the prime and target words are associated, or the type of pattern mask (e.g., immediate vs. delayed) following an ignored prime. Based on these considerations, we thought important to replicate the differential influence of WMC on ignored priming effects as a function of masking type that was recently observed by Megías et al. (2020). This was the first aim of the present research.

The second and more important goal was to investigate whether individual differences in WMC could also modulate electrophysiological (ERPs) correlates of semantic NP from single prime words.

The event related potentials (ERPs) technique provides a powerful research tool to examine the brain-electrical correlates and processes associated with NP due to their high temporal resolution (Hinojosa et al., 2009). Whereas the ERPs methodology might be a complementary dependent variable to behavioral measures (e.g., RTs and error rates) to elucidate the processes underlying NP, the attempts to obtain electrophysiological measures of NP had been relative scarce, and have produced a quite heterogeneous pattern of ERP correlates.

An early ERP component that has demonstrated to be sensitive to identity (or repetition) NP is an enhanced N200 amplitude in the ignored repetition (NP) condition, relative to a control condition. An enhanced frontal negativity is usually observed in conflict (e.g., Stroop, go/no-go) tasks when the suppression of an inappropriate response is required (e.g., Eimer, 1993; Falkenstein et al., 1999; West and Alain, 2000). Given the N200 NP effect is usually found at frontal electrode sites (e.g., Frings and Groh-Bordin, 2007; but see Daurignac et al., 2006; Hinojosa et al., 2009, for more widely distributed N200 effects), it has been interpreted as evidence for attentional inhibition (Frings and Groh-Bordin, 2007; see also Kopp et al., 1996; Kiefer et al., 1998).

The NP effect has also been associated with modulations of later ERP components as the late positive complex (LPC). Using an auditory identity-based NP task, some researchers have reported a smaller parietal positivity in the NP condition (compared to the control condition) between 450 and 600 ms post-target (e.g., Mayr et al., 2003, 2006). This NP-related LPC effect shares polarity, time course and topography with the “old/new” effect that is frequently observed in studies on recognition memory (e.g., Rugg and Doyle, 1994; Wilding, 2000). The old/new effect consists of a more positive-going ERP component that is registered particularly at parietal recording sites from 300 to 800 ms following the onset of an “old” compared to a “new” stimulus. This ERP effect has been shown to co-vary with the quality of information retrieved from episodic memory and item’s familiarity (e.g., Rugg and Doyle, 1994; Wilding, 2000). According to Mayr et al. (2003), a probe target that had previously been presented as an ignored prime distractor in a NP task, could be viewed as being “less familiar” than a “novel” target (control condition), thus resulting in an attenuated LPC associated to the NP effect.

By using a picture naming task, Behrendt et al. (2010) also reported a significantly smaller LPC amplitude in the NP condition relative to a control condition. This LPC attenuation was again interpreted in support of a memory-based account of NP (e.g., Rothermund et al., 2005). Note, however, that the NP-related reduced LPC reported by Behrendt et al. (2010) was found at *fronto-central* (and not at parietal) recording sites. The different topographies of the LPC effects could reflect different cognitive processes (e.g., Ullsperger et al., 2000). As Behrendt et al. (2010) acknowledge, the NP-related frontal LPC modulation observed in their study could also be explained in terms of a differential amount of cognitive control. Processes related to cognitive control have been associated with a late frontal ERP negativity (e.g., Ridderinkhof et al., 2004; West et al., 2004; Yeung et al., 2004). Cognitive control processes could be particularly required in a conflicting (e.g., NP) conditions, thus reducing the ERP positivity that usually indicates the completion of trial processing.

Note that a NP-related diminished posterior positivity in late time ranges (e.g., between 400 and 500 ms post-stimulus) has also been reported by some other studies, which is interpreted as a correlate of increased distractor inhibition (e.g., Gibbons and Frings, 2010; see also Gibbons, 2009). For example, Gibbons and Frings (2010) developed a variant of the flanker task, in which the prime locations slightly differed between subsequent trials. This varied-locations task was designed to minimize (or disable) anticipatory spatial prime selection, thus inducing a deeper processing of the prime distractors (i.e., their conceptual representations would be more strongly activated), as compared to a standard fixed-locations flanker task (which supposedly allowed for anticipatory inhibition of the upcoming prime distractor locations).

According to Houghton and Tipper (1994), stronger initial distractor activation should call for stronger subsequent distractor inhibition, which in turn would result in a stronger NP effect. Gibbons and Frings (2010) found that both behavioral NP and a posterior (parietal) amplitude ERP reduction in a time window of 380–430 ms were only present in the varied-location, but not in the fixed-location flanker task. The authors interpreted this NP-related ERP correlate as an increased processing negativity (see also the reversed N400 reported by Bermeitinger et al., 2008), which would be an index of the amount of activation needed for target identification. In an ignored repeated (NP) trial more activation (and effortful processing) is needed (compared to a control trial) to identify and to respond to a probe target that was previously presented as an ignored prime distractor.

Apart from these inconsistences between studies and the heterogeneity of ERP findings (which could indeed reflect the involvement of different mental processes underlying NP), note that most of attempts to measure ERP correlates of NP have used any kind of repetition (identity) priming paradigm. To our knowledge, there is but only one ERP study of semantic NP: Wagner et al. (2006), examined repetition and semantic priming effects produced by attended and ignored prime words in a lexical decision task in both patients with schizophrenia and healthy adult controls. The attended primes produced a similar behavioral and ERP pattern (N400) of priming effects in the two groups. The N400 ERP component has been widely used as an electrophysiological index of semantic processing at both the sentence and word level (Kutas and Hillyard, 1984; for a review, see Kutas and Federmeier, 2011). In conventional semantic priming paradigms, a reduced (i.e., less negative) N400 amplitude is elicited by semantically related targets, relative to unrelated targets (e.g., Kiefer, 2002). In the Wagner et al. (2006)’s study, the N400 amplitude was equally modulated by semantic context for both patients and controls. Regarding the ignored prime words, semantic NP was shown to modulate ERPs in the N400 range only in healthy controls, but not in schizophrenic patients.

It should be noted, however, that there were some remarkable dissociations between the ERP priming effects and those found on behavioral (response times) measures. Whereas the ignored primes lead to a reliable repetition NP for both controls and patients, *no behavioral semantic NP* was found at all either in patients or in control participants (Wagner et al., 2006; see pp. 206, Tables 3, 4). Thus, the N400 effects in the semantic NP condition exhibited by the control group were not functionally linked to any behavioral NP effect.

The present research was mainly aimed to explore whether higher- vs. lower-WMC participants could show not only behaviorally different semantic priming effects, but also dissociable electrophysiological correlates that were functionally linked to behavioral measures of semantic NP. To this end, we measured event-related brain activity (ERP) in individuals high and low in WMC (as assessed by their performance in complex span and attention control tasks), while they performed the same semantic single NP task as that recently used by Megías et al. (2020).

Regarding behavioral data, we expected to replicate the differential priming pattern as a function of masking type and WMC that was reported in Megías et al. (2020)’ s study: When the ignored prime was immediately followed by a persisting mask, no reliable semantic NP was expected to emerge for either group. With a delayed mask, however, variations in WMC should modulate semantic priming effects, such that only higher-WMC participants (but not those with lower-WMC) should show reliable semantic single NP (i.e., a significant three-way Relatedness × Masking Type × WMC interaction).

In the ERP recordings, we also predict differential effects for the immediate and delayed masking conditions. Research on single NP (i.e., without distractors) has consistently showed a lack of NP with an immediate and persistent mask, and some prior studies have even reported reliable facilitatory (positive) priming from ignored stimuli under this immediate masking condition (e.g., Daza et al., 2007; Wang et al., 2014). There is compelling evidence that the N400 amplitude is modulated not only by conscious, but also by unconsciously perceived words (e.g., Deacon et al., 2000; Kiefer and Spitzer, 2000; Kiefer, 2002; Kiefer and Brendel, 2006; Küper and Heil, 2009; Kiefer and Martens, 2010; Ortells et al., 2016a). It is therefore conceivable to find a conventional N400 ERP modulation (i.e., larger N400 amplitude for unrelated than for related targets) in the immediate masking condition. To the extent that the presence of an immediate mask impedes conscious identification and controlled processing of an ignored prime (or makes them more difficult), the observed N400 modulation should be similar for both higher-WMC and lower-WMC groups.

Regarding the delayed masking condition, note that no study so far has reported ERP correlates of semantic NP that are functionally linked to behavioral NP effects. For that reason, this part of our research was exploratory. But based on previous findings showing behavioral semantic NP for high-WMC, but not for lower-WMC participants (e.g., Ortells et al., 2016b; Megías et al., 2020), we expected that individual differences in WMC should modulate either early (N200) or late (LPC) (or both) ERP correlates of semantic NP.

## Materials and Methods

### Participants Screening for Working Memory and Attention Control Capacity

A sample of 200 native Spanish participants (mean age = 25.3 years, range 18–48, SD = 8.4) with normal or corrected-to-normal vision was prescreened for WM and attention control capacities, based on their performance on Spanish adaptations of automated versions of two complex verbal (Operation span-Ospan) and visual (Symmetry span-Symspan) WM tasks (e.g., Unsworth et al., 2005, 2009; see also Ortells et al., 2016b), as well as versions of the Antisaccade and Stroop conflict tasks (Kane et al., 2001; Hutchison, 2007). The presentation order of the four tasks was counterbalanced across participants (for further details of each task used in the screening phase, see Megías et al., 2020).

In the complex span WM tasks, participants must recall either the identity (Ospan) or the spatial location (Symspan) of a variable set of items (Ospan = 3–7 letters; Symspan = 2–5 squares within a matrix) in the same order in which they were presented, while performing other simultaneous task (Ospan = solving simple arithmetic operations; Symspan = making vertical symmetry decisions on visual geometric figures). The total score is the sum of items correctly recalled in the correct order without intrusions, which ranges from 0 to 75 in the Ospan, and from 0 to 42 in the Symspan WM task. In addition to obtaining independent global scores in the two span tasks for each participant, we also calculated a z-score WMC composite, by averaging z-scores for each participant across the two span tasks. Quartiles were then computed from the averaged distribution, with z-scores ranging from +0.60 and −0.40, corresponding, respectively, to the upper and lower quartiles of the 200-participants’ database.

In the Antisaccade task, participants are required to identify a briefly presented and post-masked target letter, which appears on either the same (prosaccade block) or the opposite (antisaccade block) visual field of a visual cue (an asterisk) that precedes the target (300 ms before). To make the detection of the target easier, participants are encouraged to either look away from the asterisk location, or move their eyes to the location of the asterisk in the antisaccade and prosaccade trial blocks, respectively. It is assumed that attention orienting is less automatic and more dependent on executive control in the antisaccade than in the prosaccade condition.

In the Stroop task, participants had to quickly respond to the ink color (blue, green, or red) of a central word (BLUE, GREEN, or RED), with congruent trials being much more frequent (70%) than incongruent trials (30%). These task conditions were aimed to place greater demands on working memory, such that participants should stay focused on the color naming task and avoid reading the word (e.g., Kane and Engle, 2003; Hutchison, 2007).

### Participants

Twenty-eight high (19 females) and twenty-eight low (21 females) WMC participants, who had WMC composite z-scores falling within the upper (>+0.60) and lower (<−0.40) quartiles of our 200-participants pool (see Table 1 below), were recruited for the semantic NP study, while their event-related potentials (ERPs) were recorded. Data of four participants were excluded due to excessive EEG artifacts (>60% of artifact trials; a minimum of 50% of artifact-free trials per condition was considered necessary for inclusion into the Grand Average), leaving 26 subjects in each group (High vs. Low) for behavioral and ERP analysis. These sample sizes were similar than those used by previous single-prime NP studies (e.g., Chao and Yeh, 2008; *n* = 20; Daza et al., 2007; *n* = 24; Ortells et al., 2016b; *n* = 24; Wang et al., 2014; *n* = 25; Wang et al., 2018; *n* = 24, Experiments 2–4). We further conducted a *post hoc* power analysis using G^∗^Power software 3.1.9.2 (Faul et al., 2007) to determine the statistical power of both main and interaction effects (within-between subject factors) showed in our study. With an *alpha* = 0.05, a medium effect size (*d* = 0.30) and total sample size = 52, the analysis revealed a statistical power greater than 0.99.

**TABLE 1:** Summary statistics for performance in the complex span WM tasks (Ospan, Symspan, and z-score global composite) and attentional control tasks (antisaccade and stroop congruency) by Low-WMC and High-WMC groups.

	**Low-WMC Mean (SD)**	**High-WMC Mean (SD)**	**Group differences**	**Effect Size *d***

**Span WM tasks**				
Ospan score	16.4 (6.7)	50.3 (6.6)	*t* (50) = 18.4	5.67
Symspan score	9.4 (5.7)	24.6 (4.9)	*t* (50) = 10.2	3.53
z-score composite	−1.02 (0.45)	0.98 (0.33)	*t* (50) = 18.4	5.17

**Antisaccade task**				

**Prosaccade condition**				
RT (ms)	519 (133.3)	429 (79.2)	*t* (40)^a^ = 2.9	0.82
ACC (%)	93 (9)	98 (4)	*t* (33)^a^ = 2.4	0.57
**Antisaccade condition**				
RT (ms)	702 (139.7)	544 (130,6)	*t* (50) = 4.2	1.16
ACC (%)	71 (12)	94 (6)	*t* (36)^a^ = 8.6	2.42

**Stroop task**				

**Congruent condition**				
RT (ms)	673 (178.8)	631 (175.5)	*t* (50) = < 1	–
ACC (%)	99 (1)	99 (1)	*t* (50) = < 1	–
**Incongruent condition**				
RT (ms)	833 (195.7)	678 (174.5)	*t* (50) = 2.78	0.83
ACC (%)	95 (7)	99 (3)	*t* (31)^a^ = 2.53	0.74

*All p-values < 0.05. ^a^Correction of dfs for unequal variances. The possible range of scores for Operation span and for Symmetry span tasks are 0–75 and 0–42, respectively.*

Participants were between 18 and 48 years old (*M* = 25.7, *SD* = 9.2 for high capacity; *M* = 24.4, *SD* = 8.3 for low capacity), and all of them received credit toward course requirements as compensation. All participants signed a written consent, after the nature and the consequences of the study had been explained. This research was approved by the University of Almería Human Research Ethics Committee and conducted in accordance with the Declaration of Helsinki.

### Stimuli and Apparatus

Stimulus presentation and response recordings were controlled by E-prime software (Psychology Software Tools Inc).^1^

The stimulus set was similar to that recently used by Megías et al. (2020). It consisted of 32 familiar Spanish nouns of 4–6 letters length belonging to two semantic categories (16 animals and 16 body parts). From that 32-word set, 16 were presented only as primes and 16 appeared only as targets. Half of the words from each category were randomly chosen to be presented in the immediate mask trials, while the remaining half appeared in the delayed mask condition (the assignment of words to the masking conditions was counterbalanced across participants). For each participant and masking type, the same prime and target words (four pairs from each semantic category) were presented on both related and unrelated trials (see also Megías et al., 2020). The related prime-target pairs were highly associated category members (i.e., the first ranked exemplar on both forward and backward directions, such as LION-tiger or THIGH-leg; see Callejas et al., 2003). The prime and related target words were re-paired in a pseudorandom way to create the unrelated prime-target pairs, such that the prime words from each category were followed by low associated target words belonging to the other category (e.g., LION-leg; THIGH-tiger).

**FIGURE 1:**
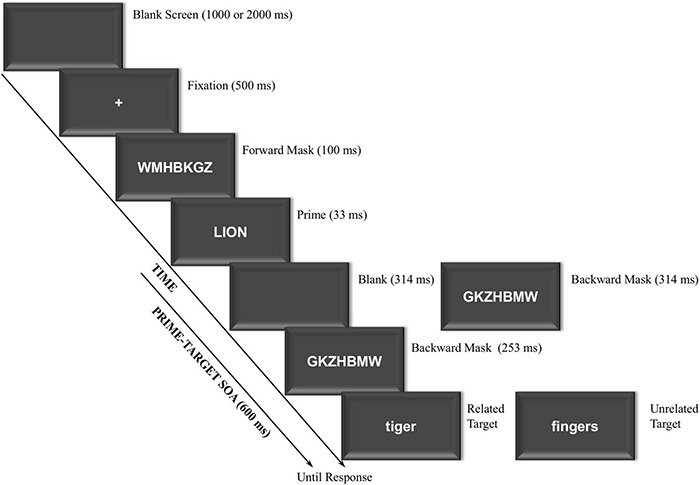
Sequence and time of events in experiment. The word stimuli shown here for related and unrelated trials have been translated from Spanish to English.

All stimuli were presented on the center computer screen at a viewing distance of approximately 60 cm, and they were displayed in white font against a black background on a 17-in CRT monitor synchronous with the screen refresh rate of 60-Hz (16.67 ms). Each trial included the following critical displays (see Figure 1): Blank screen, fixation, forward mask, prime, either backward mask or blank screen plus backward mask (depending on masking condition), and target. The blank screen was shown with a black background. The fixation display consisted of a central white cross (+) presented against on a black background. Different random strings of seven white uppercase consonants subtending a visual angle of about 2.46° wide and 0.49° high, were used as forward (e.g., WMHBKGZ) and backward (e.g., GKZHBMW) masks, respectively. The prime and target displays included of a single word presented at the center of the screen in uppercase and lowercase, respectively. Both the prime and target words subtended an averaged visual angle of about 2.21° wide and 0.49° high.

### Design and Procedure

General task instructions were displayed on the monitor and were also orally delivered. The sequence and the timing of the events were as follows (see Figure 1): (1) Blank screen presented for either 1,000 or 2,000 ms, with both durations varying randomly within the experiment to avoid anticipations; (2) Fixation display (+) presented for 500 ms; (3) Forward mask (random string of consonants) presented at the center of the screen for 100 ms; (4) Prime display containing a single uppercase word centrally presented for 33 ms, which participants should try to ignore (treating it as a distractor stimulus); (5) Either a backward mask (a different random string of consonants) presented for 567 ms until the target onset (immediate masking condition), or a 314-ms blank screen followed by a 253-ms mask (delayed masking condition) and then for the target (thus resulting in a fixed prime-target SOA of 600 ms), with the immediate and delayed masking conditions varying randomly from trial to trial; (6) Target display consisting of a single lowercase word centrally presented until response, on which participants made a categorization judgment (animal vs. body part).

This experimental procedure is identical to that used by Megías et al. (2020), except for the response keys, which were changed to be better suited for use within the context of ERP recordings (i.e., a gamepad replaced the conventional keyboard to avoid potential artifacts by body movements). Thus, on each trial participants were required to respond as fast and as accurately as possible to the target word by pressing one of two backside buttons of a gamepad with their left and right index fingers, with the mapping between categories and response keys being counterbalanced across participants. Task instructions encouraged participants to consider the prime word preceding the target on every trial as a to-be-ignored distractor stimulus.

Participants took part in a single experimental session (lasting about 35 min) consisting of 16 practice trials followed by 256 experimental trials divided into 4 consecutive blocks of 64 trials each. Half of the trials within each block were related trials, with the prime and target words being highly associated members from the same semantic category (e.g., TIGER-lion; FACE-eyes). The remaining half were unrelated trials, in which the prime and target words belonged to different semantic categories (e.g., TIGER-arm; FACE-cat). For each participant, the words presented on the immediate masking trials were always different to those presented on the delayed masking trials.

The main factors manipulated were WM Capacity (WMC), manipulated between-participants at two levels (High vs. Low-WMC), Prime-Target Relatedness (Related vs. Unrelated), and Masking Type (Delayed vs. Immediate mask). The last two factors were manipulated within-participants with a different random order for each individual. Half of the trials were “Related,” and half were “Unrelated.” Within each of these conditions, the immediate and delayed masking trials occurred equally often and in a randomized order.

After completing the categorization (priming) task, participants performed a prime visibility test to assess their awareness about the prime words presented under both immediate and delayed masking conditions. This test included 8 practice trials followed by 64 experimental trials, 32 trials for each masking condition. The sequence and timing of events were identical to those of the categorization task, with the difference that participants were now instructed to categorize the prime rather than the target stimulus. Instructions stressed accuracy over response speed. Participants were informed that the prime word could be either an animal or a body-part with an identical probability (0.50). If they were unable to categorize the prime, they were required to make the best guess without time limit.

### EEG Recording and Analysis

Participants were seated in a comfortable armchair in a dimly lit, electrically shielded room. Scalp voltages were continuously recorded from 29 active electrodes mounted in a cap (actiCAP, Brain Products, Munich, Germany) organized according to the international 10-10 system. An electrode between Fpz and Fz was connected to the ground, and an electrode between Fz and Cz was used as recording reference. Eye movements were monitored with supra- and infraorbital electrodes. Two additional electrodes were attached over the left and right mastoids so that the ERP data could be off-line re-referenced to averaged mastoids. Electrode impedance was maintained below 5 kΩ. Brain electrical signals were digitized with a sampling rate of 250 Hz (0.1–70 Hz band-pass, 50 Hz notch filter) by an AC-coupled amplifier (Brain Amp, Brain Products, Munich, Germany). After recording, data were digitally band-pass filtered (high cutoff: 25 Hz, 24 dB/octave attenuation; low cutoff: 0.1 Hz, 12 dB/octave attenuation), and segmented from 200 ms before target onset to 800 ms after target onset.

The EEG was corrected for ocular artifacts (eye movements and blinks) using independent component analysis (ICA; Makeig et al., 1997). Remaining ocular and muscular artifacts were rejected off-line in any EEG channel (maximum amplitude in the recording epoch ± 100 μV; maximum difference between two consecutive sampling points 50 μV; maximum difference of two values in the epoch 200 μV; lowest allowed activity-change 0.5 μV in successive intervals of 100 ms) and were excluded from averaging. EEG data were corrected to a 150 ms baseline prior to target onset. Finally, electrodes were re-referenced off-line to averaged mastoids. Artifact free EEG segments to trials with correct responses were averaged separately both for the four combinations of prime-target relatedness and masking conditions (with the mean percentage of EEG analyzable epochs per condition given in parentheses): Delayed masking (94.98 and 95.25% for related and unrelated conditions, respectively); Immediate masking (94.85 and 95.37% for related and unrelated conditions, respectively).

ERP effects in two time-windows were analyzed (the exact position and extension of both time windows was based on visual inspection): a negative component between 300 and 400 ms post-target onset (N400) and a late positive component between 490 and 590 ms post-target onset (LPC). Nine electrodes of fronto-central and centro-parietal scalp regions (electrodes sites: FC1/FC2, C3/C4, P3/P4, FCz, Cz, Pz), in which the N400 (e.g., Kiefer and Brendel, 2006; Kutas and Federmeier, 2011; Ortells et al., 2016a) and LPC ERP components are usually largest (e.g., Davis et al., 2003; Daltrozzo et al., 2012; Liu et al., 2014), were selected for statistical analyses. Mean amplitudes in both 300–400 and 490–590 ms time ranges were computed for each of those electrodes. Repeated measures 2 × 2 × 2 × 3 × 3 ANOVAs were performed on each time window, treating WM capacity (High- vs. Low-WMC) as a between-participants factor, and Prime-target Relatedness (Related vs. Unrelated), Masking Condition (Delayed vs. Immediate), Caudality (fronto-central, central, parietal), and Laterality (left, mid, right), as within-participant factors (*p* level of 0.05). The Geisser and Greenhouse (1959) correction was applied to all repeated measures with more than one degree of freedom, when appropriate. In order to examine the time-course of priming effects under each masking (delayed vs. immediate) condition in more detail, mean amplitudes in seven successive 100 ms epochs starting at target onset (0 ms) and running through the end of the typical LPC (600 ms) window were also analyzed.

## Results and Discussion

### Working Memory and Attention Control Tasks

Descriptive statistics (means and standard deviations) for performance in the two WM tasks (global span and z-composite scores), and the Antisaccade and Stroop attention tasks (RTs and accuracy) for both high-WMC and low-WMC groups are presented in Table 1. Participants were assigned to each WMC group on the basis of their compound global z-score in the WM tasks with no overlap.

As can be seen in Table 1, independent samples *t*-tests demonstrated that the high-WMC group outperformed the Low-WMC group in the two WM tasks, as well as in both latency and accuracies of the two attention control tasks. Note also that relative to low-WMC participants, high-WMC individuals seem to show not only a generalized task performance advantage (e.g., faster processing speed), but also an increased capacity for attentional control. Thus, the differences between the two-WMC groups appear to be greater for the more demanding conditions of the two attention tasks (e.g., antisaccade and incongruent trials). These impressions were confirmed by results of further mixed analyses of variance, in which WMC (high vs. low) was treated as a between-participants factor, and either Saccade Type (antisaccade vs. prosaccade), or Stroop Congruency (congruent vs. incongruent), as the within-participants variable.

Results from the Antisaccade task showed significant main effects for both WMC group [ACC: *F*_(1, 50)_ = 51.6, *p* < 0.001, η^2^ = 0.51; RTs: *F*_(1, 50)_ = 15.9, *p* < 0.001, η^2^ = 0.24] and Saccade Type [ACC: *F*_(1, 50)_ = 89.03, *p* < 0.001, η^2^ = 0.64; RTs: *F*_(1, 50)_ = 112.2, *p* < 0.001, η^2^ = 0.69], and of more interest, a reliable interaction between WMC and Saccade Type in both accuracy [*F*_(1, 50)_ = 48.7, *p* < 0.001, η^2^ = 0.49], and response latency [*F*_(1, 50)_ = 5.73, *p* = 0.02, η^2^ = 0.10]. This interaction revealed that the improved performance observed in the high WMC group, compared to the low one, was greater in the antisaccade condition than in the less attention demanding prosaccade condition (see Table 1).

The ANOVA on the Stroop task revealed a very similar result pattern. Namely, there were reliable main effects for both WMC [ACC: *F*_(1, 50)_ = 4.4, *p* = 0.04, η^2^ = 0.08; RTs: *F*_(1, 50)_ = 10.4, *p* = 0.02, η^2^ = 0.17], and Stroop Congruency [ACC: *F*_(1, 50)_ = 8.4, *p* = 0.05, η^2^ = 0.14; RTs: *F*_(1, 50)_ = 74.6, *p* < 0.001, η^2^ = 0.60], such that performance in the Stroop task was better on congruent than on incongruent trials. More importantly, there also was significant the interaction between WMC and Stroop congruency [ACC: *F*_(1, 50)_ = 8.4, *p* = 0.05, η^2^ = 0.14; RTs: *F*_(1, 50)_ = 10.4, *p* = 0.02, η^2^ = 0.17]. Thus, the differences in performance between high and low-WMC participants were much larger in the incongruent than in congruent trials (see Table 1).

### Behavioral Results

#### Priming Task

Trials containing incorrect responses (3.69% of total) or those with RTs falling more than 2.5 standard deviations from the overall mean RT (2.9% of trials) were removed from analyses. Mean RTs and relative frequencies of errors in percent per participant and per condition were entered in two Analyses of Variance (ANOVAs) with WM capacity (High- vs. Low-WMC) as a between-participants factor, and Masking Condition (Delayed vs. Immediate), and Prime-target Relatedness (Related vs. Unrelated) as within-subject variables. Mean RTs and mean error rate as a function of Masking condition and Relatedness for each WMC group are shown in Table 2.

**TABLE 2:** Mean (SD) reaction times (in milliseconds), and error percentages (in %) as a function of working memory capacity (Low vs. High WMC), Prime-Target Relatedness (related vs. unrelated) and masking condition (delayed vs. immediate mask).

	**Working memory capacity**	
	**Low-WMC**	**High-WMC**

**Delayed mask**		
**Related**	709 (173.2)	664 (97.2)
	3.8 (0.04)	4.1 (0.04)
**Unrelated**	737 (205.8)	637 (89.4)
	3.5 (0.04)	2.9 (0.03)

**Immediate mask**		

**Related**	705 (158.9)	648 (101.0)
	4.8 (0.05)	3.1 (0.02)
**Unrelated**	721 (158.2)	644 (94.7)
	4.2 (0.03)	3.4 (0.02)

**FIGURE 2:**
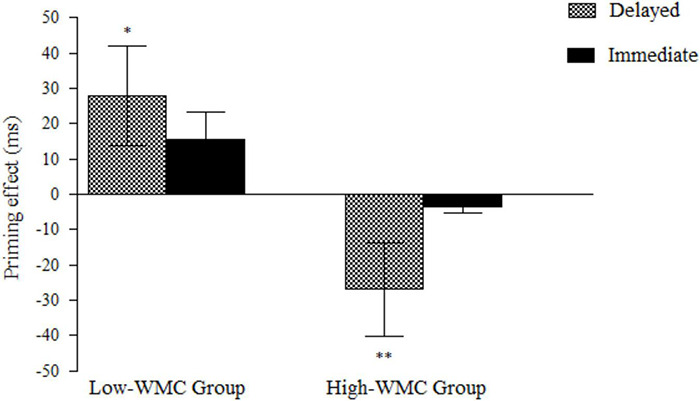
Semantic priming effects (unrelated minus related) for delayed and immediate masking for low-WMC and high-WMC participants. The vertical lines depict the standard error of mean priming scores for each condition. Statistically significant priming effects are highlighted by asterisks (*p < 0.05; **p < 0.01).

The analysis of error rates showed no significant effects (all *p*-values > 0.05). The analysis of RTs revealed a reliable interaction between WMC and Relatedness [*F*_(1, 50)_ = 12.60, *p* = 0.001, η^2^ = 0.201], such that the ignored prime words showed a reliable semantic NP effect in participants with higher WMC [−15 ms; *F*_(1, 25)_ = 5.39, *p* = 0.029, η^2^ = 0.177], whereas a positive priming (PP) effect was found in the Lower-WMC group [+22 ms; *F*_(1, 25)_ = 7.24, *p* = 0.013, η^2^ = 0.225]. Most importantly, the three-way interaction between WMC, Masking Condition, and Relatedness was reliable [*F*_(1, 50)_ = 4.076, *p* = 0.049, η^2^ = 0.075]. This triple interaction was explored with follow-up ANOVAs, separately for immediate and delayed masking conditions (see Figure 2).

When the prime word was immediately followed by a persisting pattern mask, we did not find any effect of relatedness, neither a main effect nor an interaction with WMC (all *Fs* < 1). Even when priming effects in the single conditions were compared against zero, no significant priming effects were obtained in either WMC group (see Figure 2). When the ignored single prime was followed by a delayed mask, however, we found a reliable relatedness × WMC interaction [*F*_(1, 50)_ = 14.05, *p* = 0.000, η^2^ = 0.219], which revealed an opposite priming pattern as a function of WMC, thus replicating the behavioral findings reported by Megías et al. (2020). As predicted, High-WMC participants showed a reliable NP effect [−27 ms; *F*_(1, 25)_ = 9.35, *p* = 0.005, η^2^ = 0.272], whereas a significant PP was found in the Low-WMC group [+28 ms; *F*_(1,25)_ = 5.77, *p* = 0.024, η^2^ = 0.187].

There is evidence that the size of the response-time based NP effect reliably increases under experimental manipulations leading to larger overall reaction-times (e.g., Neill and Westberry, 1987; Yee et al., 2000). Based on these findings, one could argue that the behavioral NP effect that was found in our mean RT analyses, was mainly due to very slow RTs on just a few trials in the delayed masking condition. But this does not seem the case on our research.

It has been suggested that median rather mean RTs, should be a preferred measure of central tendency in NP experiments, because of their greater resistance to single-trial outliers (e.g., Gibbons and Stahl, 2010). Accordingly, we conducted further data analyses on median RTs, which showed a very similar result pattern as that found on mean RTs. Namely, we found again a significant three-way interaction between WMC, Masking Condition, and Relatedness [*F*_(1, 50)_ = 4.79, *p* = 0.033, η^2^ = 0.087], which revealed a reliable modulation of WMC on RT priming in the delayed but not in the immediate mask. As observed in the mean RT analysis, the ignored prime followed by a delayed mask led to significant behavioral NP only in High-WMC participants [−23 ms; *F*_(1, 25)_ = 12.4, *p* = 0.001, η^2^ = 0.33], but not in the Low-WMC group (+6 ms; *F* < 1).

We also conducted an additional RT analysis, in which for each participant trials were split in fast (below-median) and slow (above-median) responses on the basis of the individual median reaction time. This analysis showed that in the High-WMC group the ignored prime followed by a delayed mask led to a reliable NP effect for slow [−35 ms; *F*_(1, 25)_ = 5.1, *p* = 0.032, η^2^ = 0.17] as well as and for fast trials [−10 ms; *F*_(1, 25)_ = 13.5, *p* = 0.001, η^2^ = 0.35]. These results provide further support for the stability and consistency of behavioral (RT-based) NP effects found in the study.

#### Prime Visibility Test

To examine participants’ prime visibility under the immediate and delayed masking trials, we computed the signal detection measure *d’* in each masking type condition for each participant. This was done by treating one level of the prime category (e.g., animal) as signal and the other level (e.g., body part) as noise (see also Kiefer et al., 2017; Wang et al., 2018). Overall discrimination for primes on delayed masking trials (*d’* = 0.12) was significantly greater [*t*(51) = 2.07, *p* = 0.044] than on immediate masking trials (*d’* = 0.02). Moreover, whereas the prime discrimination score with the delayed mask was reliably above zero [*t*(51) = 3.86, *p* < 0.001], the discrimination score with the immediate mask did not reliably deviate from zero (*t* < 1). These findings suggest that the ignored primes followed by a delayed vs. immediate mask were, respectively, above and below objective thresholds for conscious awareness.

Finally, there was no correlation between the *d*’ values of prime identification and priming scores for each participant in either group or type of mask (Immediate masking: High-WMC: *r* = −0.15, *p* > 0.46; Low-WMC: *r* = 0.15, *p* > 0.46; Delayed masking: High-WMC: *r* = 0.28, *p* > 0.17; Low-WMC: *r* = −0.22, *p* > 0.27). This finding suggests that these two indices of prime processing map on to different processes (e.g., Ortells et al., 2013, 2016a; Wang et al., 2014, 2018; see also Kiefer et al., 2019).

### Electrophysiological Results

**FIGURE 3:**
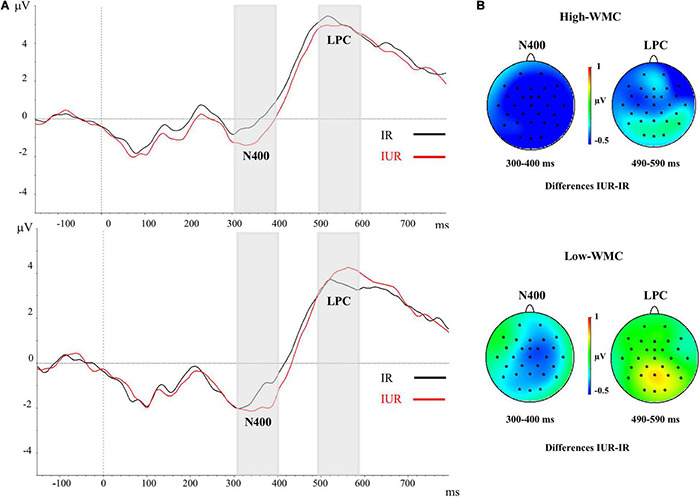
**(A)** Grand-averaged voltage data (in μV) in the Immediate Masking condition as a function of WMC and Prime-Target Relatedness (Related-R, black line, vs. Unrelated-NR, red line). As statistical analyses did not yield significant effects of the laterality and caudality factors, voltages were collapsed across the nine fronto-central and centro-parietal electrode sites (FC1/FC2, C3/C4, P3/P4, FCz, Cz, Pz). The analyzed epoch lasted from 150 ms before the onset of the target to 700 ms after target onset. Negative potentials are plotted downwards. Vertical gray shadings above the *X*-axes indicate the 300–400 ms (N400) and 490–590 ms (LPC) time windows used for statistical analysis in this and the other ERP figures. **(B)** Topographic voltage maps across the 29 electrode sites, displaying the ERP priming differences (unrelated minus related trials) in the immediate masking condition. The N400 ERP component with fronto-central and centro-parietal topography showed a global semantic priming effect in both WMC groups. No reliable LPC ERP effect was observed for neither High-WMC nor Low-WMC participants. IR, Immediate Related; IUR, Immediate Unrelated.

**FIGURE 4:**
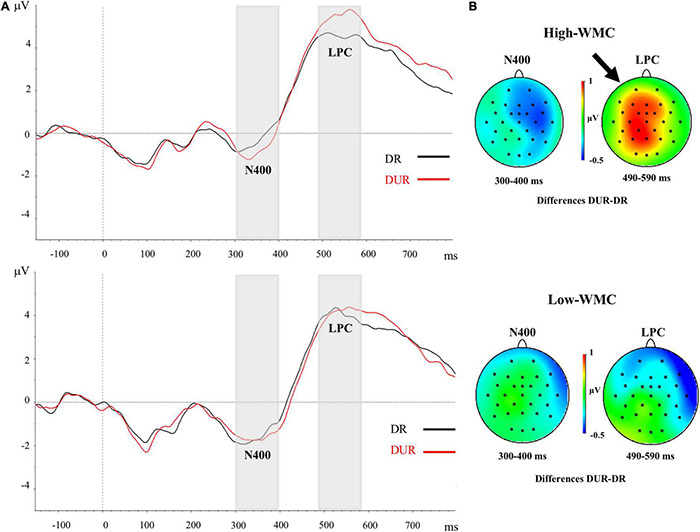
**(A)** Grand-averaged voltage data (in μV) in the Delayed Masking condition as a function of WMC and Prime-Target Relatedness (Related-R, black line, vs. Unrelated-NR, red line). As statistical analyses did not yield significant effects of the laterality and caudality factors, voltages were collapsed across the nine fronto-central and centro-parietal electrode sites (FC1/FC2, C3/C4, P3/P4, FCz, Cz, Pz). The analyzed epoch lasted from 150 ms before the onset of the target to 700 ms after target onset. Negative potentials are plotted downwards. Vertical gray shadings above the *X*-axes indicate the 300–400 ms (N400) and 490–590 ms (LPC) time windows used for statistical analysis. **(B)** Topographic voltage maps across the 29 electrode sites, displaying the ERP priming differences (unrelated minus related trials) in the delayed masking condition. The small arrowhead toward the top of the voltage map highlights the topography for the LPC correlate averaged in the time window between 490 and 590 ms, but this ERP effect was significant only in the High-WMC group. DR, Delayed Related; DUR, Delayed Unrelated.

Figures 3, 4 depict the averaged ERPs, time locked to the target onset for the related and unrelated conditions under the immediate (Figure 3) and delayed (Figure 4) masking conditions, respectively. Through the early post-target time interval (0 to approximately 300 ms) the waveforms are similar for the related and unrelated conditions under each kind of masking condition. In fact, no evidence of EEG priming effects (i.e., reliable differences in mean amplitudes between the unrelated and related conditions) for either masking type was found in the 0–300 post-target epoch (see Table 3 below).

**TABLE 3:** Time-course analyses of the ERP priming (unrelated minus related) effects for consecutive 100 ms time windows, as a function of WM capacity and masking type.

	**Low-WMC**		**High-WMC**	

	**Delayed**	**Immediate**	**Delayed**	**Immediate**
0–100	ns	ns	ns	ns
100–200	ns	ns	ns	ns
200–300	ns	ns	ns	ns
300–400 (N400)	ns	*	ns	**
400–500	ns	ns	ns	ns
500–600 (LPC)	ns	ns	**	ns
600–700	ns	ns	ns	ns

ns, p > 0.1; *p < 0.05; *p < 0.01.

### 300–400 ms Post-target Epoch (N400)

There were significant main effects for Caudality [*F*_(2, 100)_ = 78.6, *p* < 0.001, η^2^ = 0.61] and Laterality [*F*_(2, 100)_ = 12.08, *p* < 0.001, η^2^ = 0.195], but neither of both interacted with any other variable. The main effect of Prime-Target Relatedness was also significant [*F*_(1, 50)_ = 9.26, *p* = 0.004, η^2^ = 0.156], with the ERPs in unrelated trials showing a more negative voltage deflection (−1.369 μV) than in related trials (−0.896 μV; see Figures 3, 4). Thus, a reliable overall N400 ERP effect (supposedly indexing semantic prime processing) was found, which was unaffected by participants’ WMC. Although the Relatedness × Masking × WMC interaction was not significant (*F* < 1), further separate ANOVAs for each masking condition showed a reliable N400 effect only with the immediate mask [Related = −0.825 μV; Unrelated = −1.529 μV; *F*_(1, 50)_ = 14.55, *p* < 0.001, η^2^ = 0.225], but not with the delayed masking condition [Related = −0.98 μV; Unrelated = −1.20 μV; *F*_(1, 50)_ = 1.04, *p* > 0.311].

### 490–590 ms Post-target Epoch (Late Positive Complex)

**FIGURE 5:**
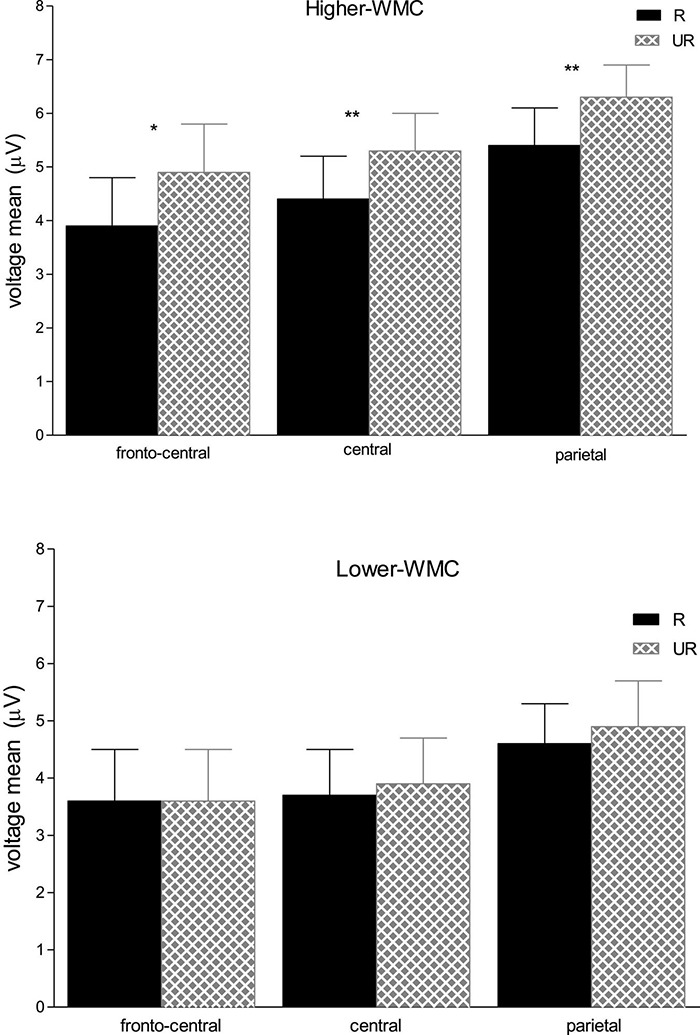
Mean ERP voltages (μV) in the 490–590 ms time interval at fronto-central (FC1/FC2/FCz), central (C3/C4/Cz), and parietal (P3/P4/Pz) electrode sites, as a function of Relatedness (Unrelated vs. Related) in the delayed masking trials, and Capacity Group (High-WMC vs. Low-WMC); error bars indicate the standard error of means, asterisks denote significant differences of Unrelated vs. Related conditions; **p < 0.01; *p < 0.05.

There were significant main effects for Caudality [*F*_(2, 100)_ = 7.19, *p* = 0.001, η^2^ = 0.13] and Laterality [*F*_(2, 100)_ = 11.01, *p* < 0.001, η^2^ = 0.18], but neither of both interacted with any other variable. There was a significant three-way interaction between WMC, Masking Type, and Relatedness [*F*_(1, 50)_ = 4.28, *p* = 0.044, η^2^ = 0.079], which revealed a differential ERP pattern depending on whether the ignored prime was followed by either a delayed mask or an immediate (and persisting) pattern mask. Further analyses of this interaction showed that the immediately masked primes produced no reliable ERP difference at this time range (all *Fs* < 1) for neither WMC group (see Figure 3). In clear contrast, when the ignored prime was followed by a delayed mask the ERPs to related targets were less positive than those to the unrelated targets (see Figure 4). Yet, this ERP difference was significant only in higher-WMC participants [Related = 4.57 μV; Unrelated = 5.48 μV*; F*_(1, 25)_ = 7.74, *p* = 0.010, η^2^ = 0.24], not in the Lower-WMC group [Related = 4.00 μV; Unrelated = 4.14 μV*; F* < 1]. Note that the LPC modulation observed in the higher-WMC group remained fairly the same irrespective of caudality (see Figure 5), as it reached statistical significance at parietal electrodes [P3/P4/Pz; *F*_(1, 25)_ = 8.9, *p* = 0.006, η^2^ = 0.27], as well as at central [C3/C4/Cz; *F*_(1, 25)_ = 7.03, *p* = 0.01, η^2^ = 0.22], and fronto-central [FC1/FC2/FCz; *F*_(1, 25)_ = 5.3, *p* = 0.03, η^2^ = 0.17] recording sites.

**FIGURE 6:**
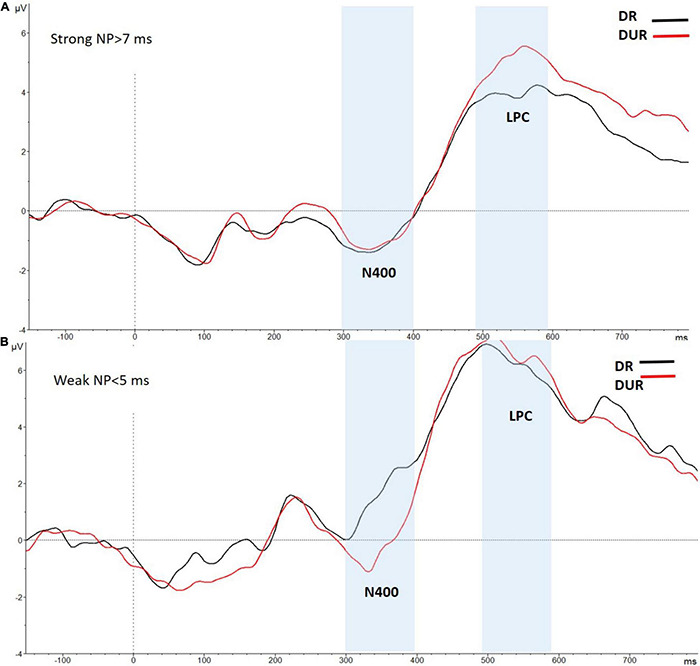
Grand-averaged voltage data (in μV) in the Delayed Masking condition as a function of Prime-Target Relatedness (Related-R, black line, vs. Unrelated-NR, red line) in the High WMC group, separately for participants showing behavioral NP (**A:** NP effect > 7 ms; *N* = 19); and participants showing behavioral facilitation or weak NP (**B:** NP effect < 5 ms; *N* = 7). Voltages were collapsed across the nine fronto-central and centro-parietal electrode sites (FC1/FC2, C3/C4, P3/P4, FCz, Cz, Pz). The analyzed epoch lasted from 150 ms before the onset of the target to 700 ms after target onset. Negative potentials are plotted downwards. Vertical gray shadings above the *X*-axes indicate the 300–400 ms (N400) and 490–590 ms (LPC) time windows used for statistical analysis.

We conducted an additional individual differences analysis, in which the ERPs data of the 26 participants in the high-WMC group were divided in two different sub-groups (for similar ERP-splitting strategy, see Frings and Groh-Bordin, 2007; Gibbons and Frings, 2010): A first participants’ subgroup (*N* = 19) showing a sizeable behavioral NP effect (>7 ms), and a second participants subgroup (*N* = 7) with either no NP or an opposite facilitation effect in the delayed masking condition. A reliable LPC modulation (reduced positivity to related relative to unrelated targets) in the time range (490–590 ms) was observed for the high-WMC subgroup who also had behavioral NP [1.30 μV; *t*(18) = 3.52, *p* = 0.02]. This related vs. unrelated ERP difference was even greater than that observed in the overall sample of participants in the high-WMC group (see Figure 6, upper-panel).

In the first 19-participants’ subgroup there also was a reliable correlation between the magnitude of the LPC priming and the size of RT NP, with a larger behavioral NP being associated with a greater LPC effect, thus supporting the claim of a functional significance of the observed LPC modulation for behavioral NP. Interestingly, this correlation was significant for both the overall (distributed across the nine recording sites) LPC ERP effect (*r* = 0.28, *p* = 0.045), and the fronto-centrally located LPC (*r* = 0.30, *p* = 0.039).

In clear contrast, the above LPC modulation was completely absent [0.15 μV; *t* < 1] for the 7-participants high-WMC subgroup who had a lack of behavioral NP in the delayed masking trials (see Figure 6, bottom-panel). Interestingly, a reliable N400 modulation (i.e., reduced positivity to the unrelated relative to related targets) in the early time range of 300–400 [−1.75 μV; *t*(6) = 3.65, *p* = 0.011] was observed for this 7- participants’ subgroup (see Figure 6, bottom-panel). This effect resembles the N400 effect showed by both high- and low-WMC individuals in the immediate masking trials. A plausible interpretation for this N400 modulation, even when the to-be-ignored prime was followed by a delayed mask (thus being clearly visible), is that despite having a high-WMC, this participants’ subgroup was unable to efficiently inhibit (or suppress) the pre-activated semantic representations of the prime. An inadequate (or absent) implementation of distractor inhibition could thus underlie the lack of RT and LPC NP effects in these individuals in the delayed mask condition. But even an ignored prime that was not-adequately inhibited, could, however, be semantically processed, thus leading to a reliable N400 ERP component (this point will further be addressed in General Discussion).

#### Time-Course Analyses

In order to better characterize the temporal profile of the ERP priming effects in the present study, we also conducted a series of further *t*-test analyses (unrelated vs. related) on the ERP data separately for the two masking conditions (Delayed vs. Immediate) in seven consecutive latency bins starting at target onset and lasting until 700 ms. Because statistical analyses had showed that Prime-target Relatedness did not interact with either Laterality or Caudality under either masking type, voltages were collapsed across the nine fronto-central and centro-parietal electrode sites. As can be seen in Table 3, no reliable ERP priming (i.e., differences in mean amplitudes between related vs. unrelated conditions) effect was observed for neither WMC group throughout the 0–300 post-target time range.

## General Discussion

There is now consistent evidence that behavioral (RT-based) semantic NP depends critically on the availability of working memory resources (e.g., Ortells et al., 2016b; Megías et al., 2020; see also Noguera et al., 2019). Note, however, that no study so far has addressed whether the individual differences in WMC could also modulate NP-related electrophysiological (ERP) correlates that were functionally linked to behavioral measures of semantic NP. This was the main goal of the present research.

To this end, we registered ERPs of participants with high and low WMC (i.e., scoring within the upper vs. lower quartiles in memory span tasks), while they performed a semantic single NP task. Participants had to categorize a probe target that was preceded by a briefly presented single prime word, which was followed either immediately or after a delay by a mask aimed to either impede or allow for its conscious identification, respectively (for a similar procedure see Daza et al., 2007; Wang et al., 2014, 2018; Megías et al., 2020).

Two kinds of relevant findings were found. Firstly, our behavioral results exactly replicated those reported by Megías et al. (2020) with a similar NP task. Namely, the ignored single prime gave rise to reliable semantic NP with a delayed, but not with an immediate mask, with the NP effect being only observed for the higher-WMC group. A second relevant finding was that the electrophysiological results also showed a modulation of both masking type and WMC on ERP priming effects. Thus, high-WMC and low-WMC participants showed a differential ERP pattern when the ignored prime was followed by a delayed mask, but not with an immediate and persisting mask. These two kinds of results will be discussed in turn.

### Modulation of Working Memory Capacity on Behavioral Semantic Negative Priming

The most relevant behavioral finding was a reliable three-way interaction between Masking Type, WMC, and Relatedness, which replicates findings reported by Megías et al. (2020) with a similar task. When the ignored prime was immediately masked, thus likely impeding participants to be aware of its identity (as the prime visibility test results suggest), no NP effect was found, irrespective of participants’ WMC. This lack of semantic NP from a single prime had previously been observed by other studies under similar masking conditions (e.g., Daza et al., 2007; Wang et al., 2014, 2018). In clear contrast, the ignored prime followed by a delayed mask led to reliable semantic NP, although this effect was only observed in higher-WMC not in lower-WMC participants (as revealed by a reliable Relatedness x WMC interaction). This latter result pattern replicates the dependence of behavioral NP on the availability of WM resources that had been reported by several previous studies with both identity and semantic NP tasks (e.g., Chao and Yeh, 2008; De Fockert et al., 2010; Ortells et al., 2016b; Megías et al., 2020; see also Gibbons and Stahl, 2010).

In addition, the RT NP in high-WMC participants in the delayed masking trials was statistically significant for both slow (above-median RT) and fast (below-median RT) response times. This demonstrates the stability and consistency of our behavioral NP effects, which are not exclusively carried by either faster or slower RTs.

Overall, the present results would be consistent with distractor inhibition accounts of NP (e.g., Tipper, 2001), as well as with executive attention theories of working memory (e.g., Engle and Kane, 2004; Hasher et al., 2007), according to which attention inhibition reflects a resource demanding (controlled) process. An increased availability of WM resources (e.g., a higher WM capacity) would be associated with a greater ability to inhibit the processing of task-irrelevant information (e.g., an ignored prime), thus explaining the emergence of reliable NP only in participants with a higher-WMC. Further evidence in support of a dependence of the semantic NP on WM resources is the finding that WMC z-composite scores for the entire sample of 52 participants did reliably correlate with the size of response-time NP (i.e., RT differences between related and unrelated trials) in the delayed mask (*r* = −0.49, *p* = 0.001), but not in the immediate mask (*r* = −0.22, *p >* 0.10) condition (for similar reliable correlations between behavioral semantic NP and WMC scores, see Ortells et al., 2016b; Megías et al., 2020).

One might wonder whether a memory-based account of NP could explain why the ignored prime followed by a delayed mask led to semantic NP only in the higher-WMC group. From an episodic-retrieval approach (e.g., Neill and Valdes, 1992; Neill et al., 1992), the NP effect would mainly reflect the incongruity between the appropriate response to a probe target and the retrieved “non-response” (or “ignore”) information to the same (or a related) stimulus when it appeared as an ignored distractor in the preceding prime episode. Some authors suggest that the finding of a reduced (or absent) NP with an increased memory load (e.g., Engle et al., 1995; Conway et al., 1999; De Fockert et al., 2010; Gibbons and Stahl, 2010) could be well explained without assuming a diminishing effect of the memory load manipulation on distractor inhibition. For example, one could argue that the NP was reduced or absent because a high memory load would impair the retrieval of prime information during probe processing, thereby reducing behavioral NP without necessarily affecting prime distractor inhibition (e.g., Lavie and Fox, 2000; see also Gibbons and Stahl, 2010).

As noted in the Introduction, the episodic-retrieval framework is not necessarily incompatible with the concept of distractor inhibition, as NP might in fact result from the episodic retrieval of prior inhibitory states of stimulus representations. For example, in the integrative approach developed by Tipper (2001), episodic retrieval process access not only tags but also inhibitory states.

However, it is highly unlikely that our behavioral NP findings exclusively relied on memory processes. If, according to a strict episodic retrieval approach, the retrieval of prior episodic traces supposedly underlying the NP is assumed to be automatic (e.g., Logan, 1988), one should expect that individuals with a Lower-WMC were as able as those with a Higher-WMC to efficiently retrieve prime information. As a consequence, the Low-WMC group should show a similar behavioral NP effect to that shown by the High-WMC group. In contrast to this prediction of episodic retrieval theory, the NP effect clearly differed between Lower- and Higher WMC individuals in our research (see also Ortells et al., 2016b; Megías et al., 2020).

The elimination of NP under the immediate masking condition could in principle be explained by an episodic retrieval theory, by assuming that presenting an immediate and persisting mask would act either (i) impeding to adequately tag the prime word as a to-be-ignored distractor, and/or (ii) interrupting (or suppressing) prime processing. However, none of these arguments seem work here. For example, in the case that participants would attend, rather than ignore, to the prime in (most or many of) the immediate mask trials, a sizeable positive priming effect should emerge in this masking condition, instead of a lack of NP, as was really the case. In the case that the immediate mask had interrupted the processing of the ignored prime, behavioral (or ERP) priming effects should be entirely absent with this masking type. Yet, a reliable N400 ERP difference between related and unrelated trials was found in the immediate masking trials (see below), thus providing clear evidence for a semantic processing of the ignored masked prime.

### Modulation of Working Memory Capacity on Electrophysiological Correlates of Priming Effects

The present research demonstrates for the first time that individual differences in working memory capacity (WMC) do reliably modulate ERP correlates of semantic priming as a function of the mask (immediate vs. delayed) that followed the ignored prime.

When the ignored prime was immediately followed by a mask, such that participants were unaware of its identity, an increased negativity to the unrelated targets (relative to the related ones) was observed at about 300–400 ms after target onset (at fronto-central and centro-parietal recording sites). This N400 modulation, usually interpreted as an ERP index of semantic processing, was significant and very similar for both High and Low-WMC participants. The observed N400 ERP priming was not associated with any behavioral (RT-based) priming effect for either WMC group. Similar dissociations between response times and ERP (N400) measures of semantic priming had been reported by several previous studies (e.g., Brown and Hagoort, 1993; Heil and Rolke, 2004; Heil et al., 2004; Kiefer and Brendel, 2006; Küper and Heil, 2009; see also Marí-Beffa et al., 2005), particularly under experimental conditions on which the prime stimuli are unattended and/or subliminally presented. Possibly, RT and ERP measures of priming occasionally capture differential aspects of cognitive processing.

The results from the prime visibility test showed that irrespective of their WMC, participants were unable to discriminate the immediately masked primes above chance, thus suggesting that the ignored primes followed by an immediate mask were below an objective threshold for conscious awareness. It appears then that the N400 modulation observed in the immediate masking trials was produced by unconsciously perceived prime words. This finding is in line with previous evidence showing that prime awareness is not a necessary condition to observe a N400 ERP modulation (e.g., Deacon et al., 2000; Kiefer and Spitzer, 2000; Kiefer, 2002; Heil et al., 2004; Rohaut et al., 2015; Ortells et al., 2016a). Yet, most of previous priming studies reporting a N400 ERP effect by subliminal primes have used a relatively short prime-target SOA (e.g., 200 ms or even lesser). The results of the present research demonstrate that it is possible to find a reliable N400 modulation even at much longer SOA intervals in some circumstances (i.e., 600 ms; see also Deacon et al., 1999, for an automatic N400 modulation at a prime-target SOA of 2000 ms).

Whereas both High- and Low-WMC participants showed a similar behavioral (lack of NP) and ERP (N400) priming pattern in the immediate mask condition, differential ERP priming effects were found for the two WMC-groups in delayed masking trials. Related (as compared to the unrelated) targets elicited a diminished positivity in a late time window (490–590 ms, LPC), which paralleled the behavioral semantic NP effect in higher-WMC participants. This LPC ERP priming effect observed with the delayed mask for the high-WMC group, already started at about 490 ms post-target. Note, however, that slow (above-median) participants’ mean RTs in that condition ranged from 743 ms (unrelated trials) to 778 ms (related-NP trials). Furthermore, in the high -WMC group the size of the behavioral NP was greater in trials with slow (above median) RTs (−35 ms) compared to trials with fast (below median) RTs (−10 ms). The LPC ERP correlate of NP does not then appear to be the consequence of the NP effect on reaction times, but it would rather be functionally linked to the processes resulting in behavioral NP.

Using different identity NP procedures and stimulus (auditory vs. visual) modalities other previous studies have also reported a NP-related LPC attenuation (e.g., Mayr et al., 2003, 2006; Behrendt et al., 2010), but this ERP effects has usually been interpreted as support for memory-retrieval theories of NP. According to Mayr et al. (2003), for example, a probe target that had previously been presented as an ignored prime distractor, could be viewed as being “less familiar” than a “novel” target (control condition), thus resulting in an attenuated LPC associated to the NP effect.

We cannot exclude the contribution of memory retrieval processes to our NP- ERP effects. As noted before, several authors assume that both attention inhibition and backward-acting retrieval processes could underlie NP (Tipper, 2001). In either case, we argue that the observed dependence of our ERP priming effects on both masking type and WMC is better explained in terms of a distractor inhibition model. This conclusion is based on several observations.

Firstly, the reduced late positivity related to the NP condition has usually been observed at posterior (parietal) electrodes (e.g., Mayr et al., 2003, 2006; but see Behrendt et al., 2010, for a frontally located LPC attenuation). In sharp contrast, a more widely distributed topography of the NP-related LPC attenuation was found in our research, in which the relatedness factor did not reliably interact with Caudality. Thus, the reduced LPC observed in the related trials (compared to the unrelated trials) for the high-WMC group in the delayed masking condition, was not confined to parietal recording sites, but it was also significant at central and fronto-central electrodes (see Figure 5). It remains possible that the different topographies of NP-related LPC modulations could indeed reflect different processes. As suggested, for example, by Behrendt et al. (2010), a frontally located NP-related LPC attenuation could also be interpreted in terms of greater cognitive control demands in the NP condition.

Secondly, LPC-like ERP effects have also reported in different attention control tasks (e.g., Kiefer et al., 1998; Jackson et al., 2001; Liu et al., 2014), with these LPC effects being interpreted as reflecting a differential ability for inhibitory control. For example, Kiefer et al. (1998) reported that response inhibition in a go/no go attention control task was associated with a sequence of distinct ERP effects, which included a later positivity (around 300–600 ms after stimulus onset) at fronto-central electrode sites, which was found in the difficult no-go task. Other studies have reported a NP-related diminished posterior positivity in late time ranges, with the effect being interpreted as an ERP correlate of increased distractor inhibition (e.g., Gibbons and Frings, 2010; see also Gibbons, 2009).

Third and even more important, the finding of a NP-related LPC modulation in the high- but not in the Lower-WMC group can be easily accommodated within a distractor inhibition framework. One crucial characteristic of inhibitory processes is their resource dependence. If the reduced LPC in delayed masking trials were an indirect consequence of a resource dependent distractor inhibition, one would then expect that ERP modulation to be mainly found in the higher-WMC group as obtained in our study. The differential LPC priming pattern of high- and low-WMC participants is, however, more difficult to explain in terms of a strict episodic retrieval model. To the extent that the retrieval of prior episodic traces supposedly underlying the NP is assumed to be automatic (e.g., Logan, 1988), one should then expect that the lower-WMC group showed similar ERP (and behavioral) priming effects to those observed in the higher-WMC group. But this was clearly not the case in our research.

Our assumption of distractor inhibition as a major source of the LPC modulation found in the delayed masking condition, receives strong support from the results of an individual differences ERP analysis in the high-WMC group. The results of these further analyses, clearly demonstrate that only those high-WMC individuals with a sizeable behavioral NP effect with the delayed mask, also showed a reliable NP-related LPC ERP attenuation. This reduced LPC component was, however, completely absent for the high-WMC participants who had no behavioral NP effect (see Figure 6). These findings provide support for the specificity and functional significance of the LPC attenuation in the delayed mask trials, as this ERP component is functionally linked to cognitive processes (distractor inhibition) underlying behavioral NP.

### Limitations and Future Directions

The NP-related LPC attenuation elicited by a semantically related (relative to an unrelated) target in the delayed masking condition was not accompanied by ERP effects in the N200 time range. Several previous studies reported a larger (frontally located) negativity of the N200 component in the NP (ignored repeated) condition, relative to a control (unrepeated) condition (e.g., Frings and Groh-Bordin, 2007; Hinojosa et al., 2009; see also Daurignac et al., 2006; Gibbons, 2006). The N200 NP effect has usually been assumed to reflect inhibitory mechanisms (e.g., response inhibition). However, a NP probe target does not require inhibition, rather inhibition might have to be resolved. From an inhibitory-based account of NP, an efficient inhibition of an ignored (distractor) prime should be associated with enhanced frontal ERP activity, given the prefrontal lobe has usually been viewed as critical for attention control (e.g., Ridderinkhof et al., 2004). An enhanced (frontal) N200 has also been observed in several attention conflict tasks, such as flanker interference (e.g., Yeung et al., 2004), stop-signal (e.g., Ramautar et al., 2006), or go/no-go tasks (e.g., Eimer, 1993), which has been interpreted as indexing response inhibition or conflict monitoring. Especially the conflict-monitoring account may be appropriate for NP, because a still-inhibited target stimulus may imply a conflict to be resolved during selection of this stimulus for action. This could be reflected in increased N200.

The question is then why no NP effect on the N200 (frontal) component was observed in our research. The NP-related N200 modulation has mostly been found in studies using a repetition (identity-based) NP paradigm, in which both the to-be-responded target and the preceding prime (and usually selected-against) distractor were the identical stimulus. This was not the case in our semantic NP paradigm. It has been suggested that the level of representation, at which attention inhibition operates, could vary as a function of task demands (e.g., Houghton and Tipper, 1994; see also Gibbons and Frings, 2010). A semantic NP task could require a relatively deeper or more advanced (e.g., abstract, categorical) level of representation, than a repetition (identity) NP task, in which distractor inhibition can operate either at an early or low feature (e.g., perceptual) level of representation. It remains possible that the NP-related N200 effects reported by previous studies were specific to NP operating at relatively early levels of processing (c.f., Gibbons and Frings, 2010). Whether individual differences in WMC could also produce a differential ERP pattern in a N200 time range when a more conventional repetition NP task is used, it remains an interesting matter for future research.

The present study was not aimed to disentangle between both persisting inhibition and episodic retrieval accounts of NP. It therefore remains open that both kind of mechanisms can contribute to the NP-related behavioral and ERP effects observed here. In either case, as a possible way to dissociate inhibitory and memory-based sources of NP, one could conduct a further ERP semantic priming experiment in which participants were instructed to either ignore or attend to a single prime word (see also Noguera et al., 2007; Ortells et al., 2016b).

To the extent that the NP-related LPC attenuation in our NP (related) condition was mainly the result of memory-based processes, we expected that a related target would lead to an *enhanced* (instead of reduced) late positivity (relative to an unrelated target), when was preceded by an attended prime. This is because the relative familiarity of a retrieved prime during probe processing, should be *greater* when this prime was previously attended than when it was ignored. In contrast, if the reduced LPC component to a related target (NP condition) would rather reflect the attention inhibition on an ignored prime, no LPC modulation should be observed in the attended prime trials, as no kind of attentional inhibition is required when participants are instructed to attend (instead of ignoring) a single prime.

In addition, the inclusion of both ignored and attended primes in a further ERP NP study, would also allow us to investigate a possible influence of attention instructions on ERP responses to the prime. Most studies addressing ERP correlates of NP, focused their analyses on ERPs elicited by the probe target. ERP waveforms during prime presentation (to which participants are usually required to overtly respond) were either not analyzed (e.g., Gibbons, 2006; Wagner et al., 2006; Hinojosa et al., 2009; Behrendt et al., 2010; Gibbons and Frings, 2010; Gibbons and Stahl, 2010), or not discussed in detail (e.g., Mayr et al., 2003, 2006; Frings and Groh-Bordin, 2007), as prime-ERP analyses reveal no differences between the priming conditions (e.g., attended related, ignored related, unrelated). This latter is indeed the expected result pattern, because at the time of prime display presentation there is complete uncertainty about the upcoming probe target type.

Yet, unlike the conventional NP task, participants in a further NP study would be instructed to either ignore or to attend to a single prime, to which no overt response is required. Under these latter task conditions, one could investigate possible ERP differences between ignored and attended primes, to provide additional support for a distractor inhibition account of NP. Some recent studies have reported a modulation of the (left-parietal) N170 ERP component elicited by the (irrelevant) prime word. This early component, assumed to reflect intentional word inhibition, was less negative when participants were instructed to explicitly ignore to the prime, as compared to an uninstructed (control) condition (Seib-Pfeifer et al., 2019). In a further analysis on prime-related ERPs in our delayed mask trials, we also found a left-parietal negativity in an early time range (extending from 150 to 250 after prime onset), which was significantly decreased in high-WMC compared to low-WMC participants. This attenuated posterior negativity in the high-WMC group, which resembles the N170 ERP reported by Seib-Pfeifer et al. (2019), did reliably correlate with the size of the behavioral NP shown by these individuals (*r* = −0.438, *p* = 0.025, *r*-critical value = −0.388). In addition, this negative component did significantly correlate with the WMC (z-composite) scores (*r* = 0.298, *p* = 0.032, *r*-critical value = 0.259). As a cautionary note, however, this prime-ERP modulation was only significant at a very limited set of recording sites (e.g., P3; P7). Whether early (and/or later) ERP waveforms evoked by a single prime word could be found as function of (i) attention instructions (ignoring vs. attending to the prime) and/or (ii) a differential availability of WM resources remain interesting issues to be addressed by future NP studies.

## Conclusion

Instructing participants to actively ignore a single prime word led to slowed response times (NP) and a diminished late ERP positivity in responses to a semantically related (as compared to an unrelated) probe target. These NP-related behavioral and ERP effects were observed (i) only when the onset of the mask following the prime was delayed (thus allowing participants to be aware of the prime), and (ii) in the group of participants with a higher-WMC. We interpreted these behavioral and ERP priming findings as being consistent with a resource dependent distractor inhibition as a major source of NP, regardless of whether the inhibition persists from the prime to the probe, or it is subsequently reinstated by a retrieval mechanism during probe processing.

The observed dependence of behavioral semantic NP on both masking type and WMC, replicates the behavioral result pattern reported by other previous studies using similar tasks (e.g., Megías et al., 2020; see also Ortells et al., 2016b).

The electrophysiological results found in the immediate masking condition replicate and extend those from several prior studies, in showing that the N400 ERP modulation, indexing semantic processing, can consistently be observed not only in the absence of prime awareness (e.g., Kiefer and Spitzer, 2000; Kiefer, 2002; Ortells et al., 2016a), but also when participants are instructed to actively ignore that unconsciously perceived prime stimulus.

The most relevant and novel finding in the present research was the NP-related LPC attenuation elicited by a semantically related (as compared to an unrelated) target in the delayed masking condition in individuals with higher WMC. This LPC modulation was specific to NP, as it emerged only under the experimental conditions leading to behavioral NP (delayed masking). It also was functionally linked to the processes resulting in RT NP effects, as the LPC attenuation appeared only in the subgroup of high-WMC participants who had a sizeable behavioral NP effect. The NP-related LPC effect was, however, completely absent in both (i) the lower-WMC group, and (ii) the high-WMC participants showing no behavioral NP in the delayed masking condition.

As far we know, this is the first time in demonstrating that a differential availability of cognitive control resources (e.g., High vs. Low-WM capacity) affects not only behavioral measures, but also electrophysiological correlates of semantic NP.

## Data Availability Statement

The raw data supporting the conclusions of this article will be made available by the authors, without undue reservation.

## Ethics Statement

This research was approved by the University of Almería Human Research Ethics Committee and conducted in accordance with the Declaration of Helsinki. The patients/participants provided their written informed consent to participate in this study.

## Author Contributions

MM and JO developed the concept and the design of the experimental work and were responsible for writing the manuscript. MM, JO, IC, CN, and MK participated in the implementation of the experimental tasks, data collection, and data analyses. All authors supervised the processes of accomplishing the study, substantially contributed to the interpretation of data, to writing and reviewing the manuscript, as well as to approving the final version of the manuscript.

## Conflict of Interest

The authors declare that the research was conducted in the absence of any commercial or financial relationships that could be construed as a potential conflict of interest.

## Publisher’s Note

All claims expressed in this article are solely those of the authors and do not necessarily represent those of their affiliated organizations, or those of the publisher, the editors and the reviewers. Any product that may be evaluated in this article, or claim that may be made by its manufacturer, is not guaranteed or endorsed by the publisher.

## Footnotes

1
https://pstnet.com/products/e-prime/
